# Meridional heat transport variability induced by mesoscale processes in the subpolar North Atlantic

**DOI:** 10.1038/s41467-018-03134-x

**Published:** 2018-03-19

**Authors:** Jian Zhao, Amy Bower, Jiayan Yang, Xiaopei Lin, N. Penny Holliday

**Affiliations:** 1 Woods Hole Oceanographic Institution, Woods Hole, MA 02543 USA; 2 Physical Oceanography Laboratory/CIMST, Ocean University of China and Qingdao National Laboratory for Marine Science and Technology, Qingdao, 266100 China; 3 National Oceanography Centre, European Way, Southampton SO14 3ZH UK

## Abstract

The ocean’s role in global climate change largely depends on its heat transport. Therefore, understanding the oceanic meridional heat transport (MHT) variability is a fundamental issue. Prevailing observational and modeling evidence suggests that MHT variability is primarily determined by the large-scale ocean circulation. Here, using new in situ observations in the eastern subpolar North Atlantic Ocean and an eddy-resolving numerical model, we show that energetic mesoscale eddies with horizontal scales of about 10–100 km profoundly modulate MHT variability on time scales from intra-seasonal to interannual. Our results reveal that the velocity changes due to mesoscale processes produce substantial variability for the MHT regionally (within sub-basins) and the subpolar North Atlantic as a whole. The findings have important implications for understanding the mechanisms for poleward heat transport variability in the subpolar North Atlantic Ocean, a key region for heat and carbon sequestration, ice–ocean interaction, and biological productivity.

## Abstract (web summary)

The oceanic heat transport is traditionally believed to be determined by the large-scale ocean circulation. New findings suggest that the energetic mesoscale processes in the Iceland Basin profoundly modulate the oceanic heat transport variability on time scales from intra-seasonal to interannual.

## Introduction

Ocean heat transport is fundamental to maintaining the earth’s energy balance. While the time-mean oceanic heat transport has been reasonably well documented using hydrographic observations and air–sea fluxes^1–3^, our knowledge of its temporal variability is less developed, in part, due to insufficient sampling of mesoscale processes in many regions. The large-scale ocean circulation, such as the Atlantic Meridional Overturning Circulation (AMOC), is found to be a big player to modulate the oceanic meridional heat transport (MHT)^4–6^. Some studies have shown, however, that mesoscale eddies also play an important role in the meridional transfer of heat. For example, observations and eddy-permitting models have indicated that eddy heat transport near the western boundary current (WBC) extensions and the Antarctic Circumpolar Current (ACC) is comparable to the time-mean heat transport^7–13^.

**Fig. 1:**
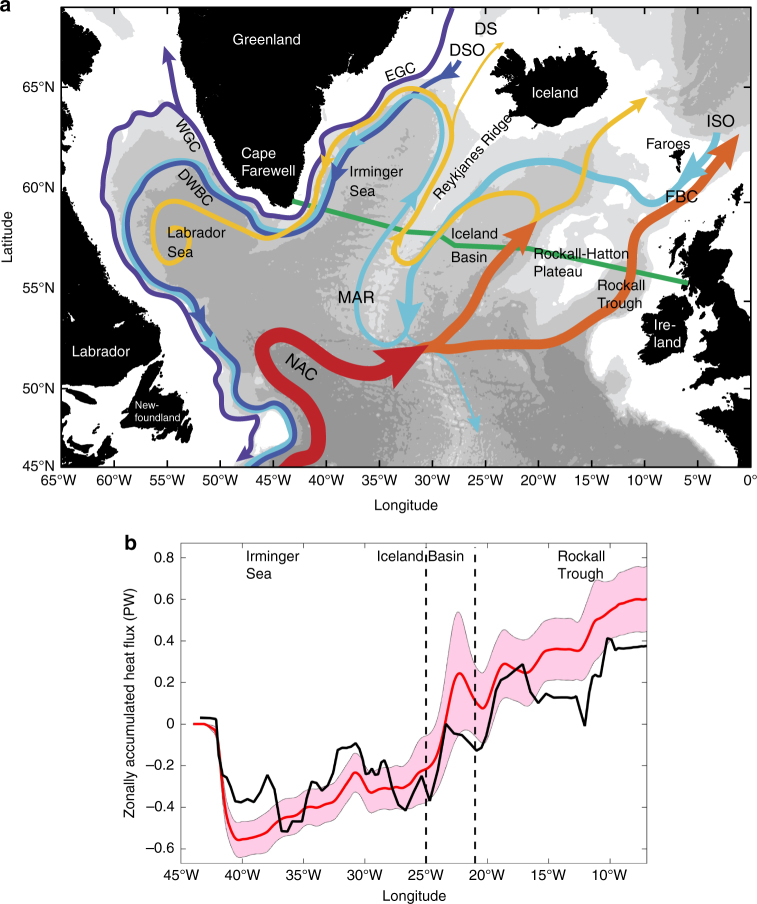
The major circulation elements and the corresponding meridional heat transport distribution in the subpolar North Atlantic Ocean.** a** The red and yellow mark the warm currents and blue and purple denote the cold currents. The map illustrates that the northward flow carries relatively warm water and southward flow generally transports colder water, leading to northward meridional heat transport in the subpolar North Atlantic Ocean. The labels are Denmark Strait (DS), Faroe Bank Channel (FBC), East and West Greenland Currents (EGC and WGC, respectively), North Atlantic Current (NAC), Denmark Strait overflow (DSO), Deep Western Boundary Current (DWBC), Iceland-Scotland Overflow (ISO), and Mid-Atlantic-Ridge (MAR). The figure is made by H. Furey, Woods Hole Oceanographic Institution and modified from Fig. 1 in Lozier et al. (2017). Green line denotes the OSNAP East section between Greenland and Scotland. **b** Meridional heat transport from surface to bottom zonally accumulated from Greenland towards Scotland along the OSNAP East line. Black solid line is the heat transport computed from in situ hydrographic observations in June 2014. Red solid line is the mean heat transport computed from 1/12 degree HYCOM simulation (1992–2014) and red shaded area represents the uncertainties measured by standard deviation. The vertical black dashed lines mark locations of the OSNAP glider transect endpoints

The Atlantic Ocean dominates the global oceanic heat transport, and its northward heat transport reaches a maximum of 1.3 PW at 26.5°N, where 1 PW = 10^15^ W (refs^2,5^). In the subpolar North Atlantic, northward moving warm waters release heat to the atmosphere and thereby are transformed into the deep and intermediate water masses that feed the deep limb of the AMOC. A transatlantic observing system (Overturning in the Subpolar North Atlantic Program, OSNAP)^14^ was initiated in summer 2014 to continuously monitor the variability of the meridional volume, heat, and freshwater transport across ~58°N and investigate the relationship between meridional transport and dense water formation. OSNAP is configured with two sections: OSNAP West extends from southern Labrador to southwestern Greenland, and OSNAP East spans from southeastern Greenland to Scotland (Fig. 1a). Previous studies have shown that almost all of the relatively warm water from southern latitudes crosses OSNAP East and leads to a mean MHT of about 0.5 PW (refs.^6,15^), while <0.05 PW crosses OSNAP West^16^ (Labrador Sea). The temporal variability for the MHT along the OSNAP East section is much greater than that along the OSNAP West^17^. In addition, the warm Atlantic-origin waters flow across the OSNAP East line and further enter the high latitudes, consequently maintaining a relatively warm climate in Northern Europe and modulating the Arctic sea ice extent^18–20^. Note that a meaningful heat transport value can only be estimated by measuring the temperature of all meridional currents in a basin (mass-conserving system). In reality, there are a net, albeit small, mass transport (about 1 Sv, where 1 Sv=10^6^ m^3^ s^−1^) across the OSNAP East and West sections, resulting from the Bering Strait throughflow to the Arctic^21^. For convenience and consistency, hereafter, we will use heat transport to refer to the temperature transport relative to 0 °C in some local regions, so that their magnitude and variability can be evaluated within the framework of basin-wide heat transport^5,6^.

The time-mean MHT in the subpolar North Atlantic is set up by the large-scale circulation, which is actually a superposition of the cyclonic gyre circulation and the AMOC (Fig. 1a). An important element of this system is the North Atlantic Current (NAC), which plays a dual role of being both the upper limb of the AMOC and the southern and eastern limbs of the subpolar cyclonic gyre. The warm waters transported by the NAC originate in the Gulf Stream, then flow northward along the western boundary east of the Grand Banks as far as about 53°N, where the NAC makes a large anti-cyclonic meander to turn eastward toward the Mid-Atlantic Ridge (MAR). East of the MAR, the main streams of the NAC head northeastward into the Iceland Basin and the Rockall Trough, and then some flow farther north into the Nordic Seas, with the remainder flowing cyclonically around the topography of the subpolar region^22–27^. It continues into the Irminger Sea on the west side of the Reykjanes Ridge (i.e., the Irminger Current) and runs parallel to the East Greenland Current (EGC) against the Greenland continental slope before flowing into the Labrador Sea^28,29^.

The contributions of different currents to the MHT are reflected in the Zonally Accumulated Heat Transport (ZAHT) over the full water column starting from the Greenland coast towards Scotland (Fig. 1b). The mean ZAHT from observations and a high-resolution (1/12°) numerical simulation suggest that the relatively cold water carried by the southward EGC and deep WBC (DWBC) leads to about −0.5 PW MHT, which is gradually compensated by the northward transport of relatively warm waters in the east. After incorporating flows in the Irminger Sea and over the Reykjanes Ridge, the ZAHT increases to −0.2 PW, indicating that these regions transport about 0.3 PW heat northward. Moving further eastward to include the Iceland Basin, the ZAHT becomes positive and increases to about 0.1–0.2 PW. Adding the Rockall Plateau and Rockall Trough, the ZAHT now becomes the total poleward heat transport and reaches the magnitude of 0.4–0.6 PW. The overall structure of ZAHT shows that the three sub-basins —Irminger Sea, Iceland Basin, and Rockall Trough—each provides about 0.3 PW northward heat transport, which more than compensates for the southward heat transport and generates a net poleward heat transport.

This study utilizes new high-resolution hydrographic and velocity observations in the Iceland Basin and an eddy-resolving model to investigate the mesoscale processes there and quantify their influence on the MHT. The observational data identifies two circulation regimes: a mesoscale eddy-like circulation pattern and the northward NAC circulation pattern. The transition between the two regimes coupled with the strong temperature front in the Iceland Basin significantly modifies the local heat transport and is the dominant source for the MHT variability on time scales shorter than 1 year. The numerical model results also suggest that these mesoscale processes produce sizable interannual variability for the MHT in the subpolar North Atlantic Ocean.

## Results

### Observations

**Fig. 2:**
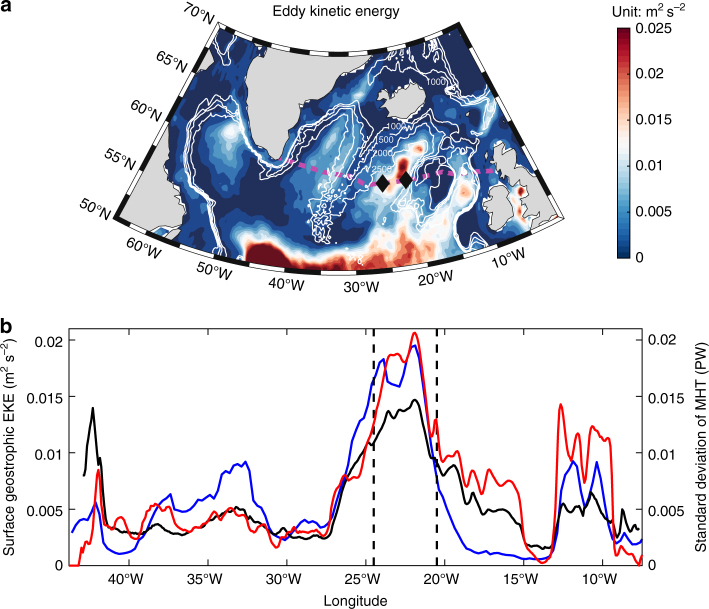
The eddy activity and meridional heat transport variability in the subpolar North Atlantic Ocean. **a** Mean surface eddy kinetic energy (EKE) from 1993 to 2015 from the satellite data. Magenta dash line represents the OSNAP East. Black diamonds denote the endpoints for the glider transect. The isobaths are illustrated by white contour lines. **b** Standard deviation of the meridional heat transport at each longitude in numerical simulation (red). The mean surface geostrophic EKE from altimeter observations (1992–2015) and numerical model (1992–2014) are displayed in blue and black, respectively. The vertical black dashed lines mark the endpoints of the glider transect, where the meridional heat transport has largest variability

Previous studies have shown that the MHT variability on seasonal to interannual time scales is more closely tied to variability in velocity or volume transport, rather than temperature^4–6^. In the subpolar North Atlantic, where the currents have a relatively strong barotropic component^27^, the surface eddy kinetic energy (EKE) provides valuable information about the spatial distribution of ocean velocity variability over the whole water column. Satellite altimetry data suggests that enhanced EKE is located in the eastern part of the subpolar region, especially in the Iceland Basin and Rockall Trough (Fig. 2), coincident with the branches of the NAC^26,30,31^. Along the OSNAP East line, the EKE maximum is co-located with the MHT variability, with the highest values located in the Iceland Basin.

**Fig. 3:**
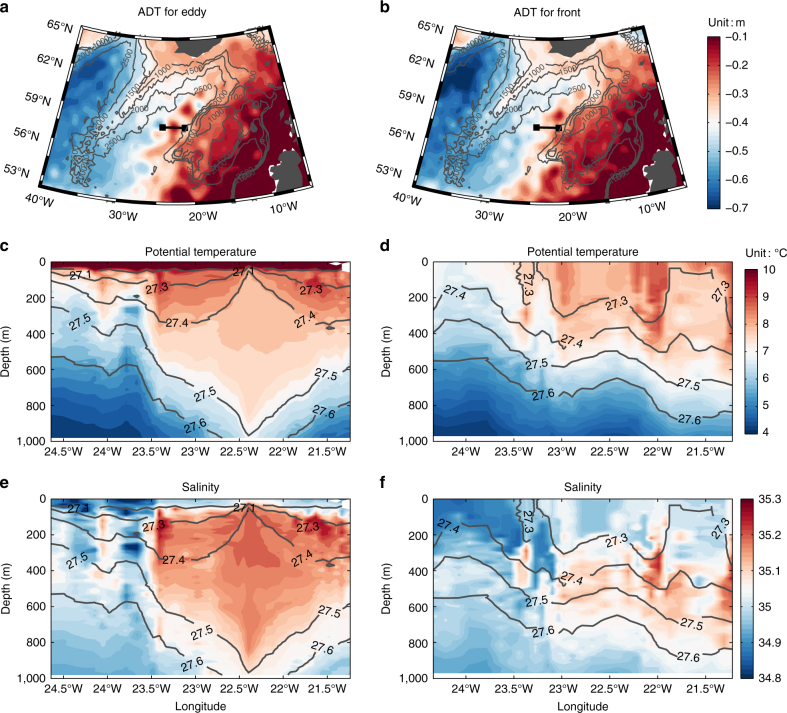
Circulation and hydrographic properties in the Iceland Basin for mesoscale eddy and frontal circulation patterns near 58°N. The left panels show the ocean state in 3–13 August 2015, for absolute dynamic topography (**a**), glider potential temperature (**c**), and glider salinity (**e**). The corresponding ocean state in 14–20 December 2016 is displayed in the right panels (**b**, absolute dynamic topography; **d**, potential temperature; **f**, salinity). Glider transect is marked by black lines in (**a** and **b**). The isobaths in (**a** and **b**) are represented by gray lines. The gray contour lines from (**c** to **f**) display the relative potential density (unit: kg m^−3^)

To investigate the potentially important role of eddies in modulating northward heat transport in this region, we successively deployed two gliders—autonomous buoyancy-driven underwater vehicles—in June and November 2015, respectively. The gliders profiled from the surface to about 1,000 m along the OSNAP East line at 58 °N between 24.5 °W and 21 °W, where both the maximum EKE and largest heat transport variability are located (Fig. 2b and Methods). Our analysis uses observed profiles of temperature, salinity, and depth-averaged velocity for the period between July 2015 and May 2016. In July 2015, a mesoscale anti-cyclonic eddy occupied the glider section (Fig. 3). The eddy had a radius of about 60 km and was characterized by a core of relatively homogenous warm and salty water (Fig. 3c, e). Similar anti-cyclonic eddies are often found in this region^32–34^. Detailed examination of the 23-year altimeter-derived absolute dynamic topography (ADT) indicates that the eddy usually occupies the glider transect for more than 2 months at a time, and that a new eddy is generated every few months, so that an eddy is apparent in the long-term mean ADT map (Supplementary Fig. 1). In October 2015, the eddy center moved to around 59°N, and a simpler frontal structure began to develop along 58°N, separating the warm, salty water to the east from the relatively cold, fresh and high oxygen water to the west. The hydrographic features associated with the eddy and front circulation patterns also project onto the velocity field and consequently affect the MHT (see Methods and Supplementary Fig. 2). A new anti-cyclonic eddy emerged in March 2016 and its characteristics were quite similar to those observed in July–September, 2015. During the observational period, the ocean circulation near the glider transect appears to be dominated by the alternation between eddy and front patterns.

**Fig. 4:**
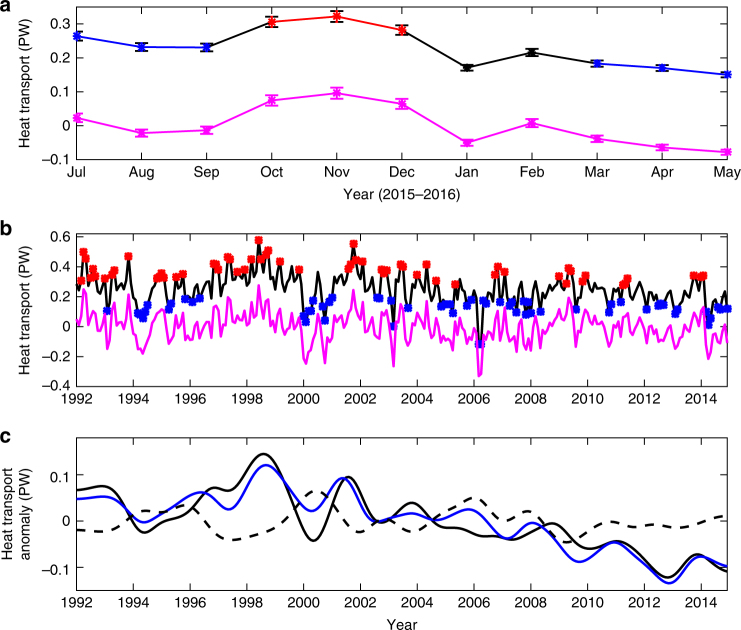
Meridional heat transport from observations and numerical model results. **a** Estimates of meridional heat transport for the upper 1,000 m across the glider section at 58°N between 24.5 and 21°W using glider observations. The estimated values, representing monthly averaged ocean state, are shown together with error bars illustrating the uncertainties due to depth-averaged velocity from the glider data. The results are separated into eddy (blue) and frontal (red) patterns. The transitional periods between eddy and front are shown in black. The magenta lines show the heat transport induced by velocity change in glider observations (**a**) and numerical model (**b**). Black line in (**b**) denotes the simulated monthly time series of meridional heat transport for the upper 1,000 m along the glider section. For comparison, the simulated mean heat transport across the glider section is 0.24 PW in the upper 1,000 m. Blue and red dots mark the eddy and front scenarios in the model. The months between those dots are transitional periods. **c** The interanual anomalies for the heat transport induced by the large-scale (black solid) and mesoscale processes (black dashed) in the Iceland Basin, respectively, are displayed. The interanual heat transport anomalies across the Iceland Basin (29–19°W, including both large-scale and mesoscale processes) is shown in blue. Unit: PW

The glider observations were used to generate monthly MHT over the top 1,000 m between July 2015 and May 2016. The mean heat transport for the monthly time series was 0.23 PW with standard deviation of 0.07 PW. Using the surface circulation pattern identified in the maps of ADT, the heat transport estimates have been separated into “eddy” (6) and “front” (3) groups (Fig. 4a). The mean heat transport is lower when the eddy is present, 0.19 PW, and increases to 0.30 PW when the eddy is replaced by a frontal pattern. These means, differing by 0.11 PW, are statistically different at the 95% confidence level using the Student's *t* test.

To further identify the underlying physical processes associated with the eddy and frontal patterns, we break the observed heat transport (*Q*_total_) down into several components using standard Reynolds decomposition, which individually represent the heat transport variability induced by temperature (*Q*_temp_), velocity (*Q*_vel_), and correlations between the two (*Q*_eddy_, see Methods). *Q*_vel_ is the dominant term and its standard deviation is 0.06 PW, very close to the variability of *Q*_total_ (0.07 PW). This indicates that the observed MHT variability is mainly driven by the ocean velocity change, which results from the alternating mesoscale eddy and frontal patterns. After examining the ADT structure in the Iceland Basin between 1992 and 2015, we conclude that the alternating mesoscale eddy and frontal structure is a common occurrence, suggesting that the mesoscale processes and the corresponding MHT variability observed by the 1-year glider observations to date are generally representative of long-term conditions.

### Model results

To put the limited observational results in a larger context, the MHT variability on different time scales is evaluated using monthly output from a high-resolution (1/12°) numerical simulation^35,36^. The simulated mean MHT across the glider transect in the top 1,000 m between 1992 and 2014 is about 0.24 PW, and its variability, in terms of standard deviation, reaches about 0.1 PW. These long-term statistics are not directly comparable with the glider observations, collected over only 1 year. However, when the simulated monthly mean MHT in the top 1,000 m is separated into eddy and front cases (Fig. 4b), we found that the maximum MHT mostly occurs under the frontal pattern when the local flow is mainly northward, and the minimum is mostly associated with the eddy structure when the local circulation is dramatically modified by the rotational currents of the eddy. The mean MHT estimates during the front and eddy patterns are 0.38 ± 0.07 and 0.11 ± 0.06 PW, respectively, yielding a difference of 0.27 PW. This difference is statistically significant at the 95% confidence level. Even if the similarity of this difference with that estimated from the gliders, 0.11 PW, is somewhat fortuitous, the tendency for higher heat transport with the frontal pattern and lower with the eddy pattern suggests that the impacts of eddy and front on the MHT variability are successfully captured by the model. Similar to the observations, the role of eddy and frontal patterns is quantified by *Q*_vel_, which has variability of 0.09 PW and is significantly correlated with *Q*_total_ (correlation coefficient is 0.97). In contrast, the variations for temperature-induced heat transport (*Q*_temp_) and eddy heat transport (*Q*_eddy_) are only 0.02 and 0.01 PW, respectively. In addition, the comparison between *Q*_vel_ and *Q*_total_ indicates that the variability of *Q*_total_ on time scales from subseasonal to interannual is mostly induced by the velocity change (i.e., *Q*_vel_).

In addition to modifying the velocity structure along the glider transect (Supplementary Fig. 2), the alternating eddy and front events can also alter the velocity field for the regions surrounding the glider track. To quantify the broader influence of mesoscale features on MHT variability, a spatial filter is applied to the numerical model output to separate the large-scale and mesoscale variability in the temperature and velocity fields. The spatially low-pass and high-pass temperature and velocity are used to compute the MHT induced by large-scale and mesoscale processes, respectively (see Methods and Supplementary Fig. 3).

Focusing first on the Iceland Basin, the standard deviation for the unfiltered monthly mean MHT across the section 29–19°W between 1992 and 2014 is 0.11 PW. The standard deviation associated with just the large-scale variability is 0.09 PW, and for the mesoscale, 0.06 PW. So it appears that the MHT variability in the Iceland Basin is almost equipartitioned between large-scale and mesoscale processes.

One might expect that the mesoscale processes dominate the MHT variability on shorter time scales, that is, <1 year, and that the larger spatial scale variability dominates on interannual and longer time scales. However, we found that mesoscale processes also contribute significantly to MHT variability on these longer time scales. To demonstrate this, we time filtered the unfiltered (i.e., the raw MHT), mesoscale, and large-scale time series of MHT for the Iceland Basin (Fig. 4c). The MHT interannual variability associated with mesoscale phenomena is about 0.03 PW, more than half of that induced by the large-scale circulation (0.05 PW). In fact, the model results show that the MHT anomalies produced by mesoscale processes are larger than that due to large-scale processes in some years (e.g., 2000 and 2006; Fig. 4c). The superposition of the individual processes at different spatial scales recovers the total MHT interannual variability in the Iceland Basin, and its standard deviation reaches about 0.06 PW. This indicates that both large-scale and mesoscale processes need to be fully resolved to accurately recover the MHT variability in the Iceland Basin, even on interannual and longer time scales.

Subpolar mesoscale processes are not limited to the Iceland Basin, and they also contribute to substantial MHT variability in the Irminger Sea and Rockall Trough (Supplementary Fig. 3). To evaluate the impact of mesoscale processes on MHT variability across the entire OSNAP East section, the unfiltered, mesoscale, as well as large-scale time series of MHT across the whole East section are obtained in a similar way to those in the Iceland Basin. Not surprisingly, the time-mean MHT (0.61 PW) is dominated by the large spatial scales (mean of 0.72 PW), and the mesoscale actually generates a southward MHT across the section (mean of −0.11 PW), induced by mesoscale activity east of Greenland (Supplementary Fig. 3).

**Fig. 5:**
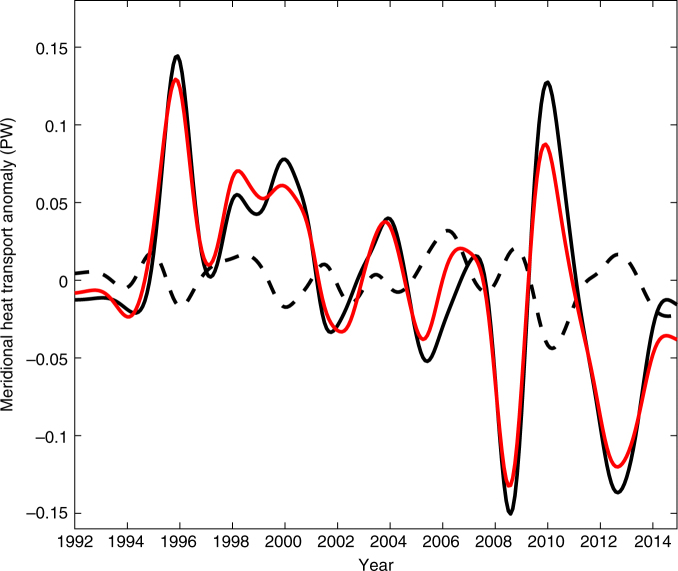
The interannual variability for the total meridional heat transport and its large-scale and mesoscale components. The interanual anomalies for the meridional heat transport along the entire OSNAP East section is shown in red. The heat transport anomalies induced by large-scale and mesoscale processes are illustrated by solid and dashed black lines, respectively. Unit: PW

Of particular interest here is how the mesoscale and large scale contribute not just to the mean, but to interannual MHT variability across the OSNAP East (Fig. 5). While the large scale dominates the total MHT interannual change, mesoscale processes also lead to sizable interannual variability, for example, in 2006 and 2010 (Fig. 5). Similar to the Iceland Basin, the velocity change on the mesoscale in space is the leading mechanism to generate the mesoscale MHT variability. Here the mesoscale MHT reflects the integral effects of all different types of mesoscale phenomena along the OSNAP East section. Its standard deviation is about 0.01 PW, or about 20% of the basin-wide MHT variability (about 0.05 PW). Therefore, the overall impact of mesoscale processes is non-negligible to the MHT variability in the subpolar North Atlantic.

## Discussion

It is widely accepted that mesoscale processes have critical consequences for the global climate through redistribution of heat and other properties in various ocean regions. For example, eddies in the tropics, the Southern Ocean, and WBC extensions were found to significantly contribute to both the time mean and the variability of the total heat transport^8,10–12,37,38^. Here, results from new in situ observations in the Iceland Basin provide a fresh perspective on the dynamics responsible for the poleward heat transport in the subpolar North Atlantic Ocean, revealing that the alternating eddy and front patterns contributes significantly to the total poleward heat transport variability on time scales from subseasonal to interannual. For the Iceland Basin, the MHT variability induced by velocity changes associated with mesoscale processes can produce about 50% of the total heat transport variability. Similarly, mesoscale processes in the Irminger Sea and Rockall Trough also play important roles in producing MHT variability. The overall mesoscale MHT variability in different sub-basins accounts for about 20% of the MHT variability across the OSNAP East section. This is different from our understanding about the mechanisms for oceanic heat transport variability, where large-scale circulation changes are believed to be the main driver^5,6^. Our results emphasize the importance of resolving mesoscale processes in observations and numerical simulations to realistically capture their roles in modulating heat transport variability in the northern North Atlantic. High-resolution observational arrays capable of capturing both large-scale and mesoscale variability, such as the OSNAP observing system (which includes moorings, gliders, Argo floats, and satellite altimetry), are needed to measure the basin-wide ocean MHT in the subpolar North Atlantic.

## Methods

### Observations

The ADT and surface geostrophic velocity fields between 1993 and 2015 were measured by the satellite altimetry. The Ssalto/Duacs altimeter products are produced and distributed by the Copernicus Marine and Environment Monitoring Service (http://www.marine.copernicus.eu). The eddy kinetic energy is defined as EKE = [(*u*′)^2^ + (*v*′)^2^]/2, where *u*′and *v*′ are derived by removing the long-term mean from the original surface geostrophic velocity. These data are used to make Figs. 2, 3 and Supplementary Fig. 1.

During the cruises in May–June 2014 and June–July 2015, conventional conductivity/temperature/depth (CTD) profiles were acquired using a SeaBird SBE-911plus pumped system, and direct velocity profiles were measured using a dual-ADCP system mounted on the CTD package (lowered ADCP (LADCP)).

Since summer 2015, G2 Slocum gliders have been jointly operated by the Woods Hole Oceanographic Institution and Ocean University of China (OUC) and serve as an important element of OSNAP to monitor the meridional volume and heat transport in the energetic Iceland Basin. The data analyzed here were collected by two gliders deployed in June and November 2015, respectively. Moving at approximately 0.2 m s^−1^, gliders “fly” through the ocean from surface to 1,000 m. In each dive-climb cycle, they navigate along a sawtooth trajectory and measure temperature, conductivity (salinity) and pressure with a Seabird pumped CTD sensor package. The horizontal sample spacing averages to be about 3 km, but near the surface and 1,000 m turnaround points, distance ranges from hundreds of meters to 6 km. The collected data are binned to profiles with vertical resolution of 1 m (Supplementary Fig. 4). The surveyed section is along 58°N with endpoints at 24.5 °W and 21°W, respectively. The section is about 200 km in length and a one-way transect is usually completed in 7–10 days.

The barotropic, or depth-averaged component of the velocity, is calculated directly from the gliders using both the glider surfacing positions and a glider flight model with calibrated parameters. This depth-averaged velocity contains all motions induced by different processes occurring in each cycle. The contributions from these processes are split into three types: geostrophic, tidal, and wind-driven Ekman currents. The motions induced by other phenomena are assumed to be errors. Therefore, the depth-averaged velocity, *v*_av_ = *v*_ek_ + *v*_tide_ + *v*_geos_.

Tidal current, *v*_tide_, is extracted using two ADCPs deployed at 300 and 500 m of the two OSNAP moorings at the western and eastern endpoints of the glider section, respectively. Each ADCP provides hourly ocean velocities in the upper ocean. The 36-h low-pass filtered velocities can be removed from the original measurements to obtain the tidal current.

The Meridional wind-driven Ekman current in the Ekman layer was derived from the zonal wind stress: $v_{{\mathrm{ek}}}\left( x,y,t \right) = \frac{{ - 1}}{{\rho fh}}\tau _{x}\left( {x,y,t} \right)$, where *τ*_*x*_, *ρ*, *h*, and *f* are the zonal wind stress, reference density, Ekman layer depth, and Coriolis parameter, respectively. The Ekman layer depth is assumed to 50 m (ref. ^39^); 1,027 kg m^−3^ is used for the reference density. Zonal wind stress comes from the daily product of ERA-Interim. The estimated wind-driven current is further weighted according to the time when the gliders stayed in the top 50 m.

The removal of *v*_ek_, *v*_tide_ from *v*_av_ is used as reference for geostrophic calculation, that is, $v_{{\mathrm{geos}}}\left( {x,z,t} \right)|_{{\mathrm{refer}}} = v_{{\mathrm{av}}} - v_{{\mathrm{ek}}} - v_{{\mathrm{tide}}}$. As mentioned above, $v_{{\mathrm{geos}}}\left (x,z,t \right)|_{{\mathrm{refer}}}$ definitely includes motions due to processes not explicitly considered here, and those are considered to be errors.

The geostrophic velocity relative to 1,000 m is computed from the density difference between pairs of density profiles according to:

$$v_{{\mathrm{geos}}}\left ({x,z,t} \right)|_{1,000} = \frac{{ - g}}{{\rho f}}\mathop {\int }\limits_{1,000}^z \frac{{\left[ {\rho _{\mathrm{e}}\left( {x,z,t} \right) - \rho _{\mathrm{w}}\left( {x,z,t} \right)} \right]}}{D}{\mathrm{d}}z.$$

where *g*, *D*, *ρ*_e_, and *ρ*_w_ are the gravitational acceleration, the distance between the density pairs, and east and west density profiles of the pairs, respectively. $v_{{\mathrm{geos}}}\left( x,z,t \right)|_{1,000}$ is further averaged over the top 1,000 m to match with $v_{{\mathrm{geos}}}\left( x,z,t \right)|_{{\mathrm{refer}}}$. The absolute geostrophic velocity is computed by adding the drift between the depth-averaged $v_{{\mathrm{geos}}}\left( x,z,t \right)|_{1,000}$ and $v_{{\mathrm{geos}}}\left( x,z,t \right)|_{{\mathrm{refer}}}$ to $v_{{\mathrm{geos}}}\left( x,z,t \right)|_{1,000}$.

The MHT is defined by $Q\left( t \right) = \mathop {\int }\limits_{x_{\mathrm{w}}}^{x_{\mathrm{e}}} \mathop {\int }\limits_{1,000}^0 \rho C_{\mathrm{p}}v(x,z,t)\theta (x,z,t){\mathrm{d}}z{\mathrm{d}}x$, where *θ* is potential temperature derived from observed temperature using SeaWater Matlab library, *C*_p_ is the specific heat of seawater, *v*(*x*, *y*, *z*, *t*) is meridional velocity, and *x*_w_, *x*_e_ are the western and eastern endpoints of the section along 58 °N, respectively. *v*(*x*, *y*, *z*, *t*) equals to the absolute geostrophic velocity for the water depth between 50 and 1000 m. In the top 50 m, the sum of absolute geostrophic velocity and Ekman current is set to *v*(*x*,*y*,*z*,*t*). Two examples of the calculated meridional velocity in the top 1,000 m along the surveyed section are shown in Supplementary Figure 2.

Uncertainties in the obtained absolute geostrophic velocity and heat transport are estimated in the following ways:

Measurement errors: All sensors were calibrated before and after the cruise. No drift was found in the conductivity measurements. According to calibration results, the measurement uncertainty of the temperature, salinity and pressure are estimated to be 0.001 °C, and 0.002 and 0.02 dbar, respectively. Incorporating them into the estimation of the geostrophic velocity relative to 1,000 m ($v_{{\mathrm{geos}}}\left( x,z,t \right)|_{1,000}$), the corresponding uncertainty is <1 cm s^−1^.

The largest uncertainty in the depth-averaged velocity is caused by the errors in the records of pitch, roll and heading when the glider is underwater. According to our calibrations, the uncertainties of pitch, roll and heading are about 10°–15°. The accuracy of GPS positions is about 10 m, but this only contributes to <0.1 cm s^−1^ error for a 6-h dive. Overall, the uncertainty in the depth-averaged velocity is about 1 to 2 cm s^−1^, which is consistent with other glider observations^40^.

Temporal variability not observed by gliders: It took 7–10 days for a glider to completely survey the 200 km long section; therefore, the variability due to the processes on the time scales shorter than 7–10 days can induce uncertainties. Observed currents from ADCPs are used to estimate the variability on time scales shorter than the period of each complete glider transect. They are taken as the uncertainties induced by the time variability not observable in glider surveys.

Errors in the meridional velocity calculations: Tidal currents were assumed to be uniform and barotropic in the surveyed region. According to the analysis using the two ADCPs deployed in the endpoints of the glider section, their difference is <1 cm s^−1^ on the tidal frequency. We thus take this number to be the uncertainties associated with the predicted tidal currents.

The wind-driven Ekman transport is assumed to be uniformly distributed in the Ekman layer, which is assumed to be 50 m. The assumptions are imperfect because observations found that the wind-driven Ekman currents have spiral-like structure and are strongly surface-trapped^41^. However, during a 6-h dive, gliders only took several minutes in the top 50 m. Therefore, the errors induced by the wind-driven Ekman current are negligible.

We also noted that there are non-Ekman ageostrophic currents, such as the motions induced by the sub-mesoscale processes near the eddy edge. These motions are irregularly distributed in space and time, so their overall impacts on the density profiles of the geostrophic velocity calculation are assumed to be small.

### Numerical simulation

The numerical simulation was performed using the eddy-resolving high-resolution (1/12°) HYbrid Coordinate Ocean Model (HYCOM). The model domain spans from 28 °S to 80 °N and was configured originally by Xu et al.^35^ The initial state was from the experiment E026 in Xu et al.^36^ where monthly climatological forcing from the European Center for Medium-Range Weather Forecasts reanalysis (ERA40) was used to spin up for 25 years. Starting from model year 25 in E026, our HYCOM simulation is further spun up for 25 years using the daily National Centers for Environmental Prediction (NCEP) Climate Forecast System Reanalysis (CFSR) data. After spin-up, the model was integrated from 1992 to 2014 forced by daily data NCEP CFSR data. The daily model outputs are used to construct the monthly mean fields that are analyzed in this study.

The 1/12° HYCOM simulations were found to successfully reproduce both the long-term mean and variations of the subpolar North Atlantic circulation, particularly the AMOC, the boundary currents in the Labrador Sea and the NAC^36^. As shown in Figs. 1 and  4c, the monthly time series of total poleward heat transport across the OSNAP East line between Greenland and Scotland has a mean value of about 0.6 PW and a standard deviation of 0.14 PW, with a minimum of 0.3 PW and maximum of 1.0 PW. These numbers are in line with the estimates using synoptic trans-basin hydrographic measurements near similar latitudes^6,15^. Near the glider transect in the Iceland Basin, an anti-cyclonic eddy can be found in the model mean surface height between 1992 and 2014 (Supplementary Fig. 5a), which is quite similar to the satellite ADT results (Supplementary Fig. 1). The EKE in the model is calculated in the same way as for the altimetry observations. The simulated mean EKE pattern also resembles well the main features in the satellite altimetry data (Fig. 2 and Supplementary Fig. 5b). The simulated meridional velocity associated with anti-cyclonic eddy composite in the Iceland Basin is shown in Supplementary Fig. 2 and its vertical structure agrees well with the in situ observations. Based on these comparisons, we conclude that the eddy-resolving HYCOM has a reasonably good skill not only in simulating the basin-wide features but also eddies in the Iceland Basin.

Mesoscale eddies in the Iceland Basin are detected in numerical results following the algorithm developed by Nencioli et al.^42^. The Nencioli algorithm consists of four constraints: first, a reversal of the meridional velocity (*v*) along an east–west section; second, a reversal of the zonal velocity (*u*) along a north–south section; third, a local minimum of the velocity magnitude at the eddy center; and last, a constant sense of rotation along the four quadrants of the eddy. The eddy scenarios in the Iceland Basin are defined using the criteria that the eddy boundary falls within the glider section. In order to identify the frontal structure, anomalies of monthly total meridional volume transport across the glider section are calculated. The standard deviation of the anomalies is 5.4 Sv (1 Sv = 10^6^ m^3^ s^−1^). The frontal structures are assumed to be established when the anomalies are positive and are larger than the standard deviation of 5.4 Sv. The eddy and frontal structures are marked in Fig. 4. Their corresponding sea surface height patterns are shown in Supplementary Fig. 6.

### Reynolds decomposition

To reveal the physical process for the MHT variability, standard Reynolds decomposition is used to separate the heat transport into several components: *Q*_total_ = *Q*_vel_ + *Q*_temp_ + *Q*_eddy_, where the left side is the heat transport and right side is the heat transport induced by velocity, temperature, and eddy, respectively.

$$Q_{{\mathrm{total}}} = \mathop {\int }\limits_{x_{\mathrm{w}}}^{x_{\mathrm{e}}} \mathop {\int }\limits_h^0 \rho C_{\mathrm{p}}v(x,z,t)\theta (x,z,t){\mathrm{d}}z{\mathrm{d}}x,$$

where *h* is the depth to integrate heat transport.

Velocity and potential temperature are decomposed as follows: $v= \bar v + v\prime$ ; $\theta = \bar \theta + \theta \prime$, where overbar denotes time average and prime refer to the fluctuating part with respect to the time mean. Therefore,

$$Q_{{\mathrm{vel}}} = \mathop {\int }\limits_{x_{\mathrm{w}}}^{x_{\mathrm{e}}} \mathop {\int }\limits_h^0 \rho C_{\mathrm{p}}v\prime (x,z,t)\bar \theta (x,z,t){\mathrm{d}}z{\mathrm{d}}x,$$

$$Q_{{\mathrm{temp}}} = \mathop {\int }\limits_{x_{\mathrm{w}}}^{x_{\mathrm{e}}} \mathop {\int }\limits_h^0 \rho C_{\mathrm{p}}\bar v(x,z,t)\theta \prime (x,z,t){\mathrm{d}}z{\mathrm{d}}x,$$

$$Q_{{\mathrm{eddy}}} = \mathop {\int }\limits_{x_{\mathrm{w}}}^{x_{\mathrm{e}}} \mathop {\int }\limits_h^0 \rho C_{\mathrm{p}}v\prime (x,z,t)\theta \prime (x,z,t){\mathrm{d}}z{\mathrm{d}}x.$$

### Spatial filter

In order to separate the large-scale and mesoscale features, a spatial Butterworth filter with a cutoff length scale of 10° in longitude (about 600 km) is applied to the velocity and temperature field of the monthly HYCOM results along the OSNAP East section. The cutoff length scale is determined by the spatial scale for the zonal shift of the NAC and eddy diameters in the Iceland Basin, which is estimated in the satellite altimetry maps. The low-pass spatially filtered velocity and temperature are defined as large-scale process. The variables for the mesoscale process are obtained by removing the low-pass filtered from the original model outputs and are named as high-pass filtered dataset. The unfiltered (i.e., the original), low-pass and high-pass spatially filtered variables are used to compute the MHT for the total, large-scale and mesoscale processes, respectively.

In addition, the time series for the three different MHTs are further split into interannual and short time scales (intra-seasonal to seasonal). This is achieved by applying a temporal Butterworth filter with a cutoff length of 2 years to all the time series. The interannual changes for the MHT induced by large-scale and mesoscale processes are exhibited in Supplementary Fig. 3.

### Code availability

The source codes for HYCOM can be downloaded online (https://hycom.org/hycom/source-code).

### Data availability

Observations collected by gliders and synoptic ship surveys are archived at OSNAP (http://www.o-snap.org/observations/data/). The satellite altimeter products are distributed by the Copernicus Marine and Environment Monitoring Service (http://www.marine.copernicus.eu). The data that support the findings of this study are available from J.Z. upon reasonable request.

## Electronic supplementary material

Supplementary Information

## References

[1] Ganachaud A, Wunsch C (2000). Improved estimates of global ocean circulation, heat transport and mixing from hydrography data. Nature.

[2] Trenberth KE, Caron JM (2001). Estimates of meridional atmosphere and ocean heat transports. J. Clim..

[3] Talley LD (2003). Shallow, intermediate, and deep overturning components of the global heat budget. J. Phys. Oceanogr..

[4] Biastoch A, Böning CW, Getzlaff J, Molines JM, Madec G (2008). Causes of interannual–decadal variability in the meridional overturning circulation of the midlatitude North Atlantic Ocean. J. Clim..

[5] Johns WE (2011). Continuous, array-based estimates of Atlantic Ocean heat transport at 26.5°N. J. Clim..

[6] Mercier H (2015). Variability of the meridional overturning circulation at the Greenland-Portugal OVIDE section from 1993 to 2010. Prog. Oceanogr..

[7] Wunsch C (1999). Where do ocean eddy heat fluxes matter?. J. Geophys. Res..

[8] Jayne SR, Marotzke J (2002). The oceanic eddy heat transport. J. Phys. Oceanogr..

[9] Gille ST (2003). Float observations of the Southern Ocean. Part II: eddy fluxes. J. Phys. Oceanogr..

[10] Phillips HE, Rintoul SR (2000). Eddy variability and energetics from direct current measurements in the Antarctic Circumpolar Current south of Australia. J. Phys. Oceanogr..

[11] Volkov DL, Lee T, Fu LL (2008). Eddy-induced meridional heat transport in the ocean. Geophys. Res. Lett..

[12] Bishop SP, Watts DR, Donohue KA (2013). Divergent eddy heat fluxes in the Kuroshio Extension at 1438–1498E. Part I: Mean structure. J. Phys. Oceanogr..

[13] Dong C, McWilliams J, Liu Y, Chen D (2014). Global heat and salt transports by eddy movement. Nat. Commun..

[14] Lozier S (2017). Overturning in the Subpolar North Atlantic Program: a new international ocean observing system. Bull. Am. Meteorol. Soc..

[15] Lumpkin R, Speer K (2007). Global ocean meridional overturning. J. Phys. Oceanogr..

[16] Pickart RS, Spall MA (2007). Impact of Labrador Sea convection on the North Atlantic meridional overturning circulation. J. Phys. Oceanogr..

[17] Li F, Lozier MS, Johns WE (2017). Calculating the meridional volume, heat and freshwater transports from an observing system in the Subpolar North Atlantic: observing system simulation experiment. J. Atmos. Ocean Technol..

[18] Häkkinen S, Rhines PB, Worthen DL (2011). Warm and saline events embedded in the meridional circulation of the northern North Atlantic. J. Geophys. Res..

[19] Vellinga M, Woods RA (2002). Global impacts of a collapse of the Atlantic thermohaline circulation. Clim. Change.

[20] Zhang R (2015). Mechanisms for low-frequency variability of summer Arctic sea ice extent. Proc. Natl. Acad. Sci. USA.

[21] Serreze MC (2006). The large-scale fresh water cycle of the Arctic. J. Geophys. Res..

[22] Bower AS (2002). Directly measured mid-depth circulation in the northeastern North Atlantic Ocean. Nature.

[23] Flatau M, Talley L, Niiler PP (2003). The North Atlantic Oscillation, surface current velocities, and SST changes in the subpolar North Atlantic. J. Clim..

[24] Lavender KL, Owens WB, Davis RE (2005). The mid-depth circulation of the subpolar North Atlantic as measured by subsurfacefloats. Deep Sea Res. I.

[25] Sarafanov A (2012). Mean full-depth summer circulation and transports at the northern periphery of the Atlantic Ocean in the 2000s. J. Geophys. Res..

[26] Chafik L, Rossby T, Schrum C (2014). On the spatial structure and temporal variability of poleward transport between Scotland and Greenland. J. Geophys. Res. Oceans.

[27] Daniault N (2016). The northern North Atlantic Ocean mean circulation in the early 21st century. Prog. Oceanogr..

[28] Holliday N, Bacon S, Allen J, McDonagh E (2009). Circulation and transport in the western boundary currents at Cape Farewell, Greenland. J. Phys. Oceanogr..

[29] Våge K (2011). The Irminger Gyre: circulation, convection, and interannual variability. Deep Sea Res. Part I.

[30] White MA, Heywood KJ (1995). Seasonal and interannual changes in the North Atlantic subpolar gyre from Geosat and TOPEX/Poseidon altimetry. J. Geophys. Res..

[31] Volkov DL (2005). Interannual variability of the altimetry-derived eddy field and surface circulation in the extratropical North Atlantic Ocean in 1993–2001. J. Phys. Oceanogr..

[32] Martin AP, Wade IP, Richards KJ, Heywood KJ (1998). The PRIME eddy. J. Mar. Res..

[33] Read JF, Pollard RT (2001). A long-lived eddy in the Iceland Basin 1998. J. Geophys. Res..

[34] Shoosmith DR, Richardson PL, Bower AS, Rossby HT (2005). Discrete eddies in the northern North Atlantic as observed by looping RAFOS floats. Deep Sea Res. Part II.

[35] Xu X, Schmitz WJ, Hurlburt HE, Hogan PJ, Chassignet EP (2010). Transport of Nordic Seas overflow water into and within the Irminger Sea: an eddy-resolving simulation and observations. J. Geophys. Res. Oceans.

[36] Xu X (2013). On the currents and transports connected with the Atlantic meridional overturning circulation in the subpolar NorthAtlantic. J. Geophys. Res. Oceans.

[37] Roemmich D, Gilson J (2001). Eddy transport of heat and thermocline waters in the North Pacific: a key to interannual/decadal climate variability?. J. Phys. Oceanogr..

[38] Rhein M (2011). Deep water formation, the subpolar gyre, and the meridional overturning circulation in the subpolar NorthAtlantic. Deep Sea Res. Part II.

[39] Rio MH, Hernandez F (2003). High-frequency response of wind-driven currents measured by drifting buoys and altimetry over the world ocean. J. Geophys. Res..

[40] Todd RE, Rudnick DL, Davis RE (2009). Monitoring the greater San Pedro Bay region using autonomous underwater gliders during fall of 2006. J. Geophys. Res..

[41] Price JF, Weller RA, Schudlich RR (1987). Wind-driven ocean currents and Ekman. Transp. Sci..

[42] Nencioli F, Dong C, Dickey T, Washburn L, McWilliams JC (2010). A vector geometry based eddy detection algorithm and its application to a high-resolution numerical model product and high-frequency radar surface velocities in the Southern California Bight. J. Atmos. Ocean Technol..

