# Author Correction: Meridional heat transport variability induced by mesoscale processes in the subpolar North Atlantic

**DOI:** 10.1038/s41467-018-04809-1

**Published:** 2018-06-14

**Authors:** Jian Zhao, Amy Bower, Jiayan Yang, Xiaopei Lin

**Affiliations:** 1 Woods Hole Oceanographic Institution, Woods Hole, MA 02543 USA; 2 Physical Oceanography Laboratory/CIMST, Ocean University of China and Qingdao National Laboratory for Marine Science and Technology, Qingdao, 266100 China

Correction to: *Nature Communications* 10.1038/s41467-018-03134-x, published online 19 March 2018

The original version of this Article omitted the author N. Penny Holliday from the National Oceanography Centre, European Way, Southampton SO14 3ZH, UK.

Consequently, the following was originally omitted from the Acknowledgements: ‘N.P.H. and the JR302 cruise were funded through the UK Natural Environment Research Council programmes UK OSNAP (NE/K010875/1), RAGNARRoCC (NE/K002511/1) and the Extended Ellett Line (National Capability)’. The corrected version of the Acknowledgements also removes the following from the original version: ‘The Zonally Accumulated Heat Transport in observation was calculated by N.P.H. from the National Ocean Center, United Kingdom’.

Additionally, the following was originally omitted from the Author Contributions: ‘N.P.H. calculated the observed Zonally Accumulated Heat Transport along the OSNAP east section’.

This has been corrected in both the PDF and HTML versions of the Article.

