# Supplementary material for: Meridional heat transport variability induced by mesoscale processes in the subpolar North Atlantic
Source: Nat Commun. 2018 Mar 19;9:1124. doi: 10.1038/s41467-018-03134-x (PMC5859275; doi:10.1038/s41467-018-03134-x)
Supplement: Supplementary file 1 — Supplementary Information [file 41467_2018_3134_MOESM1_ESM.docx]

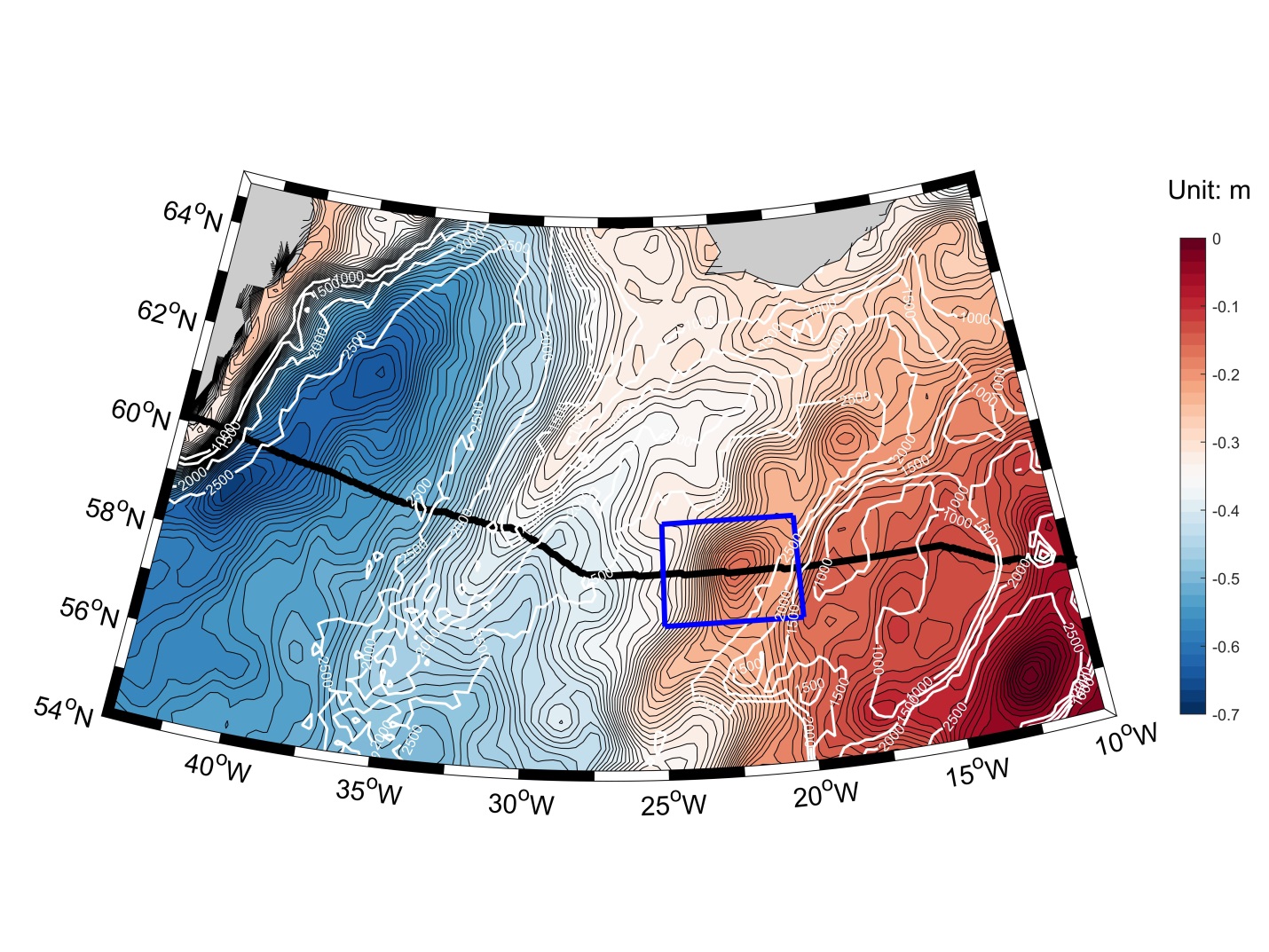


Supplementary Figure 1. **Mean Absolute Dynamic Topography created using the satellite data between 1993 and 2015.** Black line denotes the OSNAP East. The region within the blue box displays an anti-cyclonic mesoscale eddy structure, suggesting that the eddy in the Iceland Basin can affect the long-term mean circulation. Isobaths are shown in white lines.


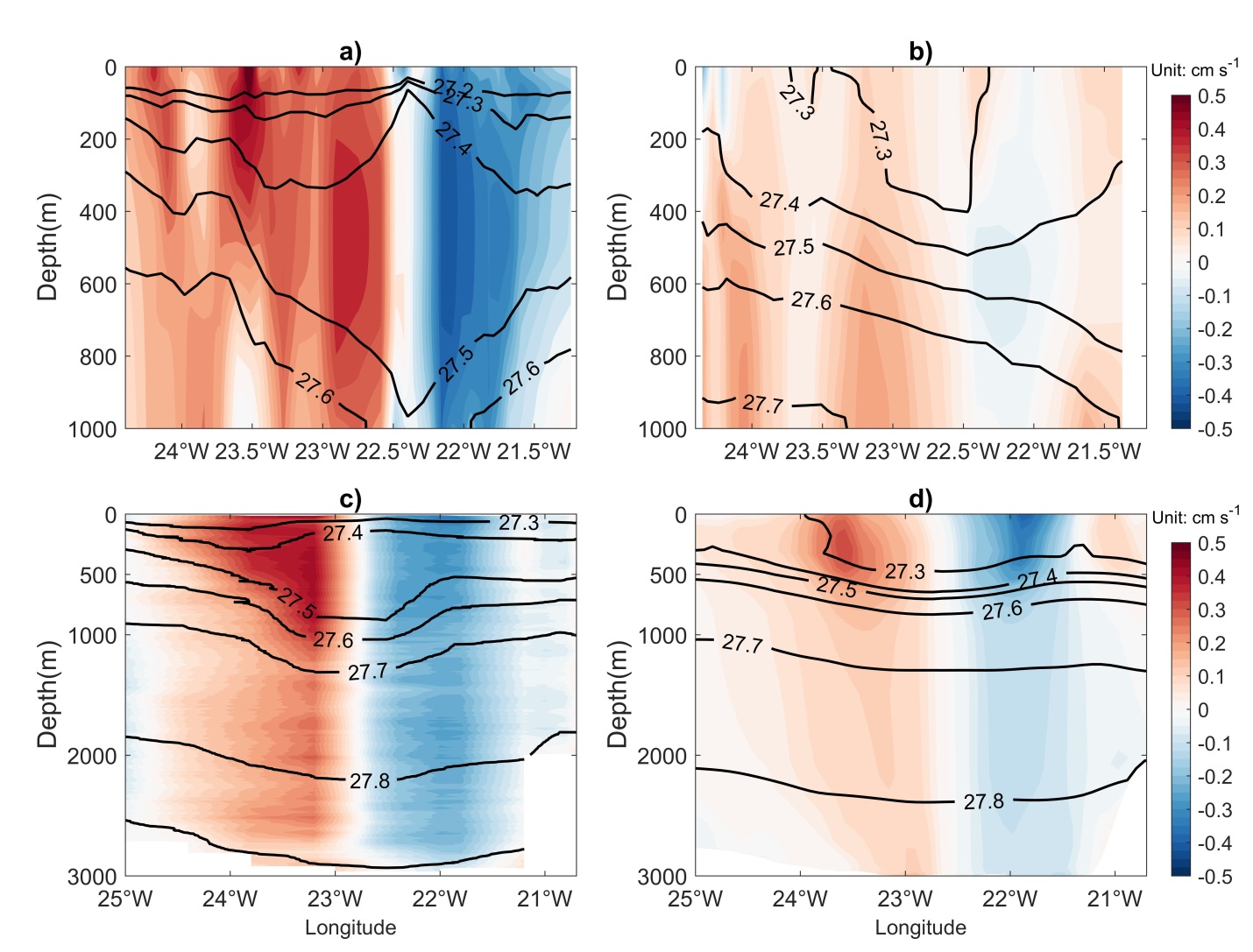


Supplementary Figure 2. **The observed meridional velocity for the eddy and front circulation patterns and the simulated eddy velocity by the numerical model**. Meridional velocity for the periods between 23 July-2 August, 2015 (a, eddy event) and between 21 and 28 December, 2016 (b, frontal structure) derived from the glider observations. c), The meridional velocity between 29 June and 1 July, 2015 is measured by a dual-ADCP system [lowered ADCP (LADCP)], whose data were processed with Visbeck/Lamont-Doherty Earth Observatory (LDEO) version 9 software (Visbeck 2002). d), meridional velocity for the anti-cyclonic eddy constructed from composite eddy events in HYCOM simulation. This demonstrates the capability of HYCOM to reasonably reproduce the observed eddy structure. Unit: m s^-1^. Black lines are the contours of potential density relative to zero pressure.


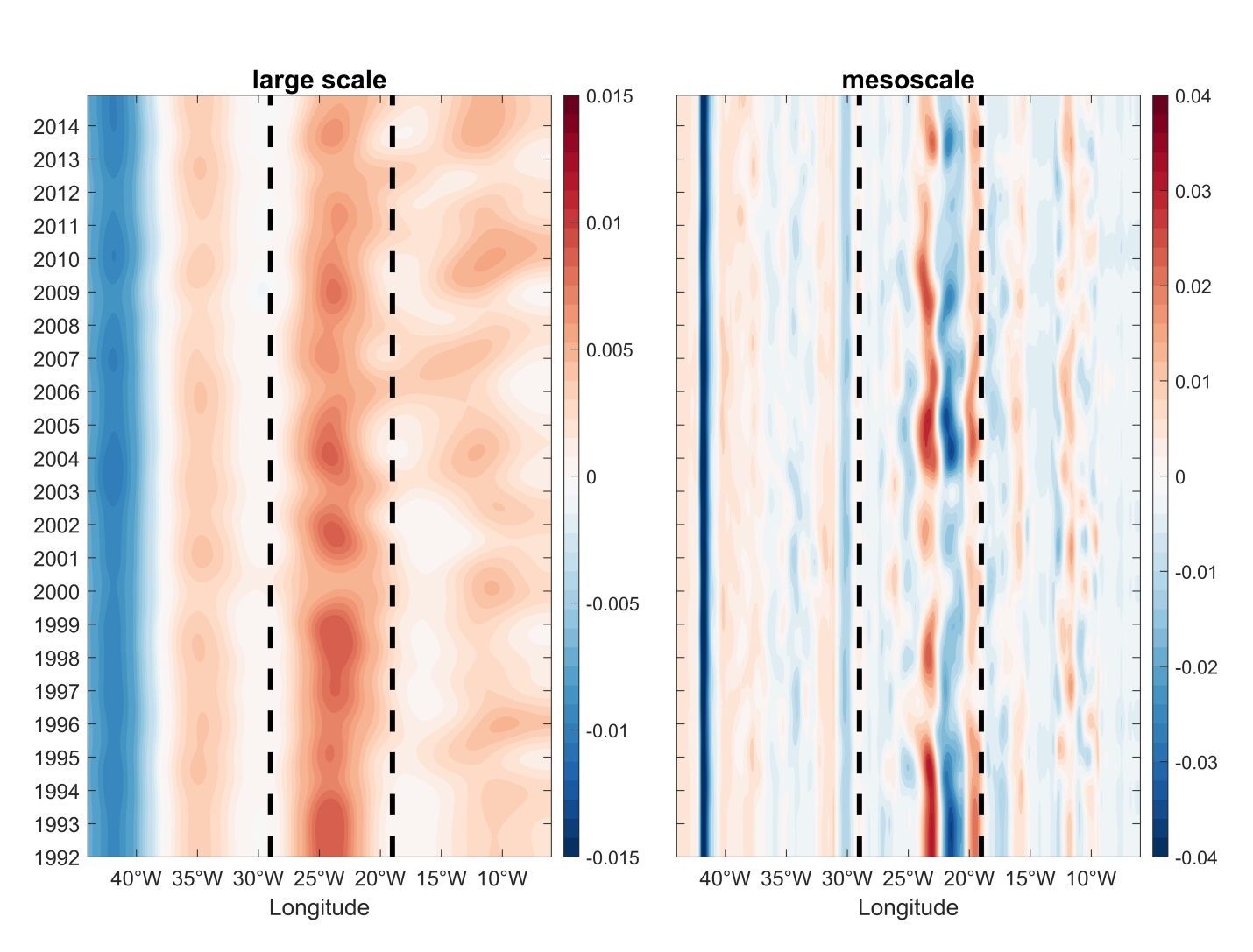


Supplementary Figure 3. **Interannual anomaly for the MHT per unit longitude along the OSNNP East line connecting Greenland and Scotland**. The MHT variability induced by the large scale and mesoscale processes is shown on left and right, respectively. Unit: PW. Black dash lines denote the boundaries of the Iceland Basin (2$9^{\circ}$Wand 19$^{\circ}$W).


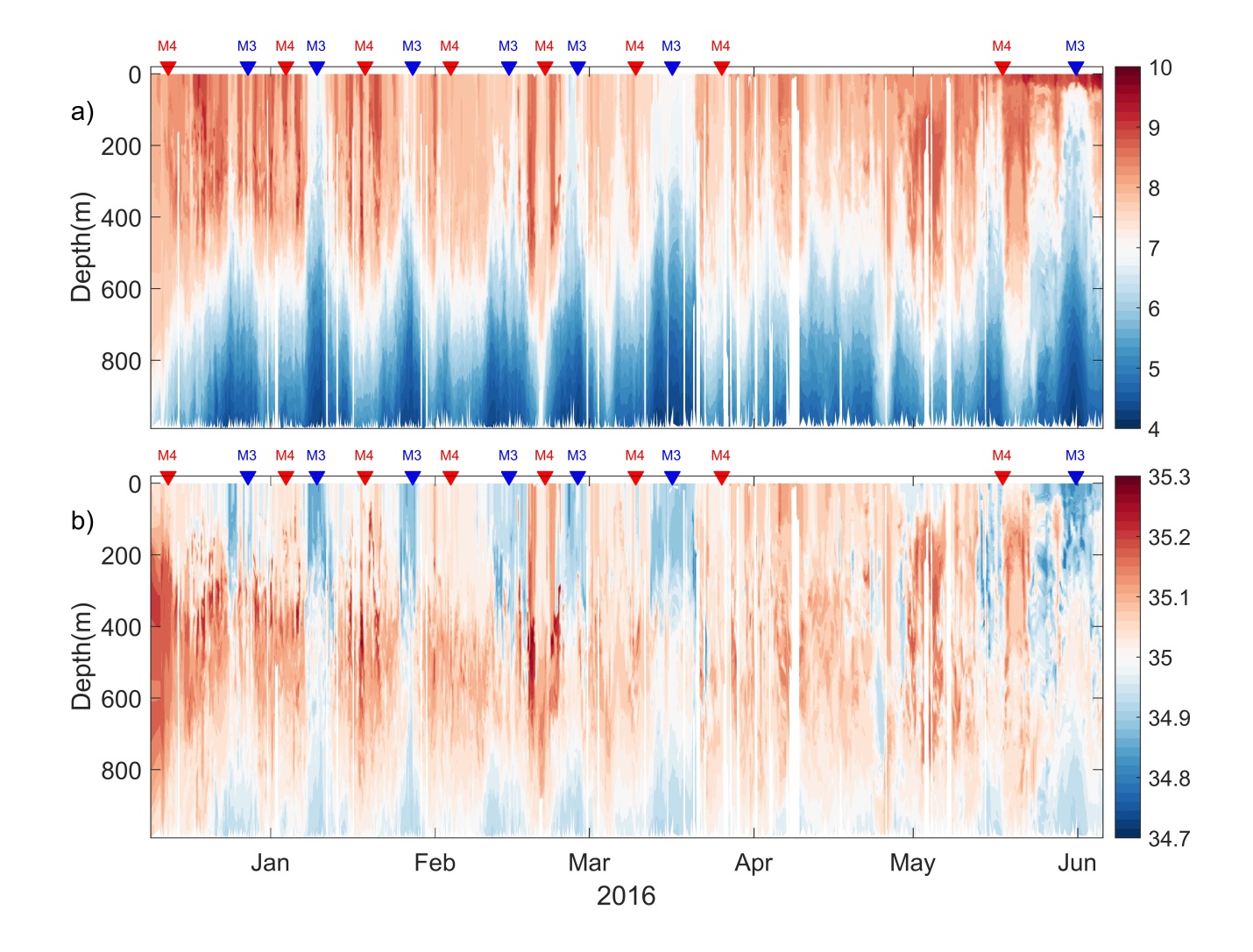


Supplementary Figure 4. **The glider observations.** Time series of Temperature (Unit:°C) and Salinity sampled by the glider between 19 December 2015 and 5 June 2016 are shown in a) and b), respectively. The red and blue triangles represent the east (warm) and west (cold) endpoints of the sampling section.


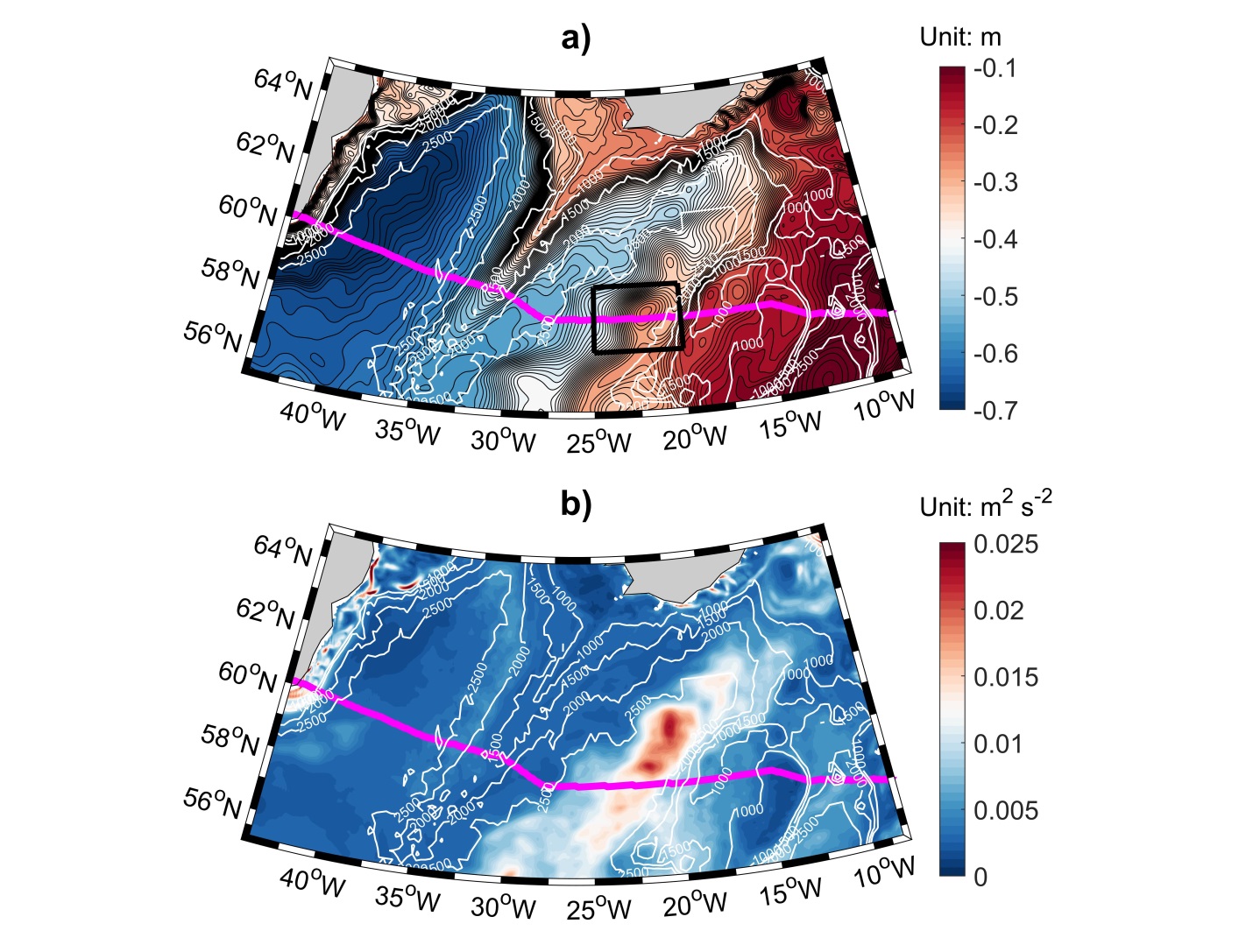


Supplementary Figure 5. **Mean sea surface height and surface Eddy Kinetic Energy (EKE) simulated by the numerical model.** a) Mean sea surface height between 1992 and 2014 from HYCOM simulation. The overall structure in the subpolar North Atlantic and the eddy-like pattern within the black box are quite similar to the satellite altimetry Absolute Dynamic Topography (Supplementary Fig. S1). b) Mean EKE between1992 to 2014 from HYCOM simulations. The most striking feature is the enhanced EKE located in the Iceland Basin, which agrees well with the satellite data (Fig.2). Magenta lines denote the OSNAP East section. Isobaths are shown in white lines.


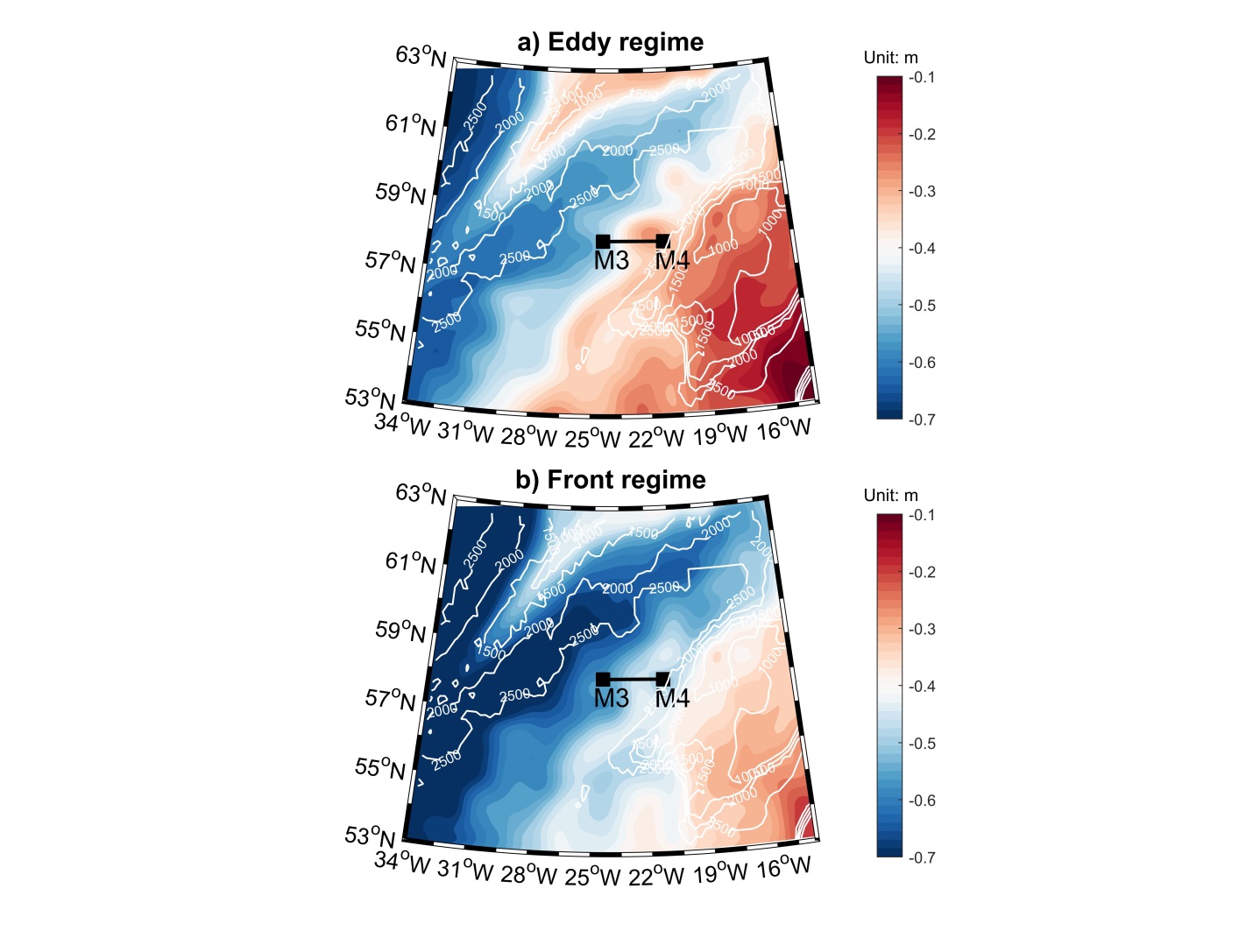


Supplementary Figure 6. **Mean sea surface height pattern in the eddy and frontal structures from the numerical simulation.** The eddy (a) and frontal (b) patterns are obtained by averaging the sea surface height during the eddy and front events in the HYCOM results. Black line denotes the glider transect whose end points are labeled with M3 and M4, respectively. Isobaths are shown in white lines.
